# Recent advances in the management of anal cancer

**DOI:** 10.12688/f1000research.14518.1

**Published:** 2018-09-28

**Authors:** Matthew M. Symer, Heather L. Yeo

**Affiliations:** 1 New York-Presbyterian Hospital/Weill Cornell Medicine, Department of Surgery, 525 East 68th Street, New York, NY 10065, USA

**Keywords:** anus neoplasms; carcinoma, squamous cell; neoplasms; antineoplastic agents; radiation

## Abstract

Anal cancer is a rare condition, although its incidence has been increasing over the past several decades, particularly in women. The majority of anal cancers are squamous cell cancers and are linked with human papilloma virus (HPV) infection. Recent work in HPV basic science has delineated the mechanism by which the virus leads to the development of anal cancer. With widespread availability of an HPV vaccine since 2006, vaccination has become an important strategy for anal cancer prevention. However, in the US, there remain no guidelines for anal cancer screening. Treatment of anal cancer is dictated largely by accurate staging, which is generally accomplished with a combination of physical exam, magnetic resonance imaging, computed tomography, and positron emission tomography. Chemoradiation remains the mainstay of treatment for most patients, with surgery reserved for salvage therapy. Recent trials have identified the optimal use of available chemotherapeutics. Exciting developments in immune therapies targeting HPV oncoproteins as well as therapeutic vaccines may soon dramatically change the way patients with anal cancer are managed.

## Introduction

The understanding and treatment of anal cancer has evolved over the past several decades. This has become particularly important as the incidence of anal cancer has increased. At an epidemiologic level, the human papilloma virus (HPV) vaccine has offered the possibility of primary prevention of cancer development. For those diagnosed with anal cancer, multimodal therapy with chemotherapy and radiation is the cornerstone of anal cancer treatment, with surgery generally reserved for those who have progression of disease despite chemoradiation. In the future, emerging therapies such as immunotherapy may become particularly important.

## Discussion

### Epidemiology

Anal cancer is a rare condition, although over the past decade its incidence has increased, in particular among women. Overall, for the past decade, anal cancer incidence has been increasing by 2.2% each year. Currently, anal cancer constitutes 0.5% of all cancer diagnoses in the United States ^1^. The development of anal cancer is strongly associated with human HPV infection, with 91% of anal cancers attributed to the virus ^2^. Sexual practices which increase the risk of HPV transmission such as anal receptive intercourse or multiple sexual partners, as well as a history of vulvar, vaginal, or cervical dysplasia or cancer, are also associated with anal cancer. The risk of anal cancer also increases with age, smoking, and immunosuppression such as in HIV/AIDS or transplant immunosuppression.

Of all anal cancers, 85% are of squamous cell histology. Anal intraepithelial neoplasia (AIN) is a precursor lesion to anal cancer and is classified according to a unified system applied to squamous cell cancers of the epithelium. This classification includes two main categories: low-grade squamous intraepithelial neoplasia (LSIL; corresponding to AIN I) or high-grade squamous intraepithelial neoplasia (HSIL; corresponding to AIN II or III) ^3^. The dichotomy of high versus low grade is an important distinction that dictates further management.

### Pathogenesis

HPV, a double-stranded DNA virus, is the most common sexually transmitted virus of the anogenital tract, and the development of anal cancer is strongly linked to HPV infection. Based on NHANES data from 2013–2014, 42.5% of US adults aged 18–59 years tested positive for genital HPV (based on penile or vaginal swab) ^4^. Most who are infected by HPV remain asymptomatic and become test negative without intervention, but a small number will develop dysplasia ^5^. Numerous HPV genotypes have been identified, with HPV-16 and -18 being the most strongly linked with the development of anal cancer. According to CDC estimates, these two subtypes alone account for 79% of all anal cancers ^2^.

Two HPV viral proteins, E6 and E7, are closely linked to oncogenesis in squamous epithelia. E6 binds to the host cell p53 and E7 binds to host retinoblastoma. By preventing apoptosis and causing cell-cycle arrest, these proteins contribute to the progression to cancer ^6, 7^. However, this step is necessary but not sufficient for malignant transformation. E6 and E7 also contribute to genomic instability, which further enhances oncogenesis ^8^.

### Prevention

As with all sexually transmitted infections, prevention of infection with HPV is an important management principle. HPV is highly transmissible via any skin-to-skin contact, and barrier methods such as condoms protect only the portion of skin covered. Penetrative intercourse is not necessary for HPV inoculation, and self-inoculation events have been reported ^9^.

A quadrivalent HPV vaccine was FDA approved in 2006, covering the two most prevalent genotypes in genital warts and the two most prevalent genotypes in cervical cancer. More recently, in 2014, a nonavalent vaccine was approved and is now widely available ^10^. While these vaccines have the potential to dramatically reduce the incidence of anal cancer, they are underutilized ^11^. In the United States, only about half of eligible adolescents aged 13 to 17 years have received one or more doses of any HPV vaccine ^12^. It will likely take years to see the effect of the vaccine, as the median age at diagnosis of anal cancer is 61 ^1^.

In the last several years, there has been growing interest in the use of the HPV vaccine as adjuvant treatment in individuals with AIN ^13^. In one retrospective review of the quadrivalent vaccine in HIV-negative men who have sex with men (MSM), with a history of HSIL, the rate of HSIL recurrence was halved ^14^.

### Screening

The US Preventive Services Task Force has not established formal recommendations for the screening of AIN or anal cancer. Although screening with digital anorectal examination is recommended in HIV-infected patients, many anal lesions may not be palpable. The HIV Medical Association of the Infectious Diseases Society of America recommends screening via anal cytology in MSM, HIV-infected individuals with genital warts, women who practice anal receptive intercourse, and women with a history of cervical atypia ^15^. The New York State Department of Health recommends screening of all HIV-infected adults ^16^.

Screening for individuals at risk of anal cancer should include anal cytology and anoscopy. Those with a past medical history conferring increased risk as well as those with cytology consistent with LSIL or HSIL should undergo high-resolution anoscopy ^17^.

### Initial workup

**Figure 1.**
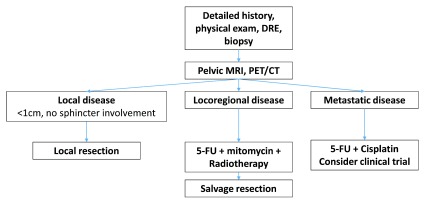
Typical approach to the diagnosis, management, and workup of anal cancer. 5-FU, 5-fluorouracil; CT, computed tomography; DRE, digital rectal examination; MRI, magnetic resonance imaging; PET, positron emission tomography.

Workup begins with a detailed history and physical exam, including complete gynecologic exam in women. Further workup is focused on accurate staging ( Figure 1). Tissue biopsy is performed for diagnosis, and excisional biopsy should be avoided if the mass is large or infiltrating, as this can delay chemoradiation ^18^. Our practice is to perform locoregional staging with pelvic magnetic resonance imaging (MRI) and systemic staging with computed tomography (CT) of the chest, abdomen, and pelvis (with contrast) and positron emission tomography (PET). Most anal cancers are PET avid, and PET is particularly useful for identifying positive inguinal or pelvic lymph nodes ^19, 20^. In one study, PET/CT altered staging in 23% of patients ^21^. However, PET/CT alone is not an appropriate staging strategy.

### Treatment

Multimodal therapy remains the mainstay of treatment for anal cancer. In recent years, these therapies have evolved and been continually refined as new classes of agents have become available. Initial treatment is reliant on accurate staging ^22^.

For small, well-differentiated lesions (<1 cm) that do not involve the sphincters, particularly those of the perianal region, local excision can be utilized with or without the addition of chemoradiotherapy. However, for most patients, chemotherapy and radiation remain the primary treatment modalities. This consists of mitomycin, 5-fluorouracil (5-FU), and an initial course of 45 Gy of radiation given over 5 weeks. An additional dose of 9–14 Gy can be provided to patients with persistent disease, larger tumors (T3/T4), or node positivity.

Several trials have examined the optimal regimen for patients with local or locally advanced disease. ACT II was a two-by-two factorial design, phase III trial comparing cisplatin with mitomycin, with both arms receiving 5-FU and radiation ^23^. This was given with or without maintenance chemotherapy. ACT II results showed that cisplatin was associated with similar response rates and toxicity. Of note, however, the cisplatin arm included an induction phase and there was an overall prolonged treatment duration which may have contributed to reduced efficacy. Conversely, long-term follow-up of the RTOG 98-11 trial showed better disease-free and overall survival with mitomycin compared to cisplatin ^24^. Based on these findings, 5-FU plus mitomycin remains the preferred chemotherapy in most patients. As an alternative, capecitabine may also be used in place of 5-FU ^25^.

For patients with metastatic disease, cisplatin-based chemotherapy combined with 5-FU has been established as optimal treatment. As with many other malignancies, immunotherapy has become a promising area of research in anal cancer. Based on the observation that HPV-mediated oncoproteins upregulate checkpoint molecules to evade cell-mediated cytotoxicity, several studies of anti-PD-1 monoclonal antibodies have been performed. A phase II trial of nivolumab in patients with refractory metastatic anal cancer achieved a response in 24% of patients ^26^. Promising results were also seen in a phase I trial of pembrolizumab in PD-L1-positive patients with treatment failure and locally advanced or metastatic disease.

Aside from the management of small lesions, surgery is reserved for the management of complications and as salvage therapy. This may include recurrence, as well as patients with persistent or progressive disease. Some data suggest that as many as 30% of patients with anal cancer will progress to abdominopelvic resection (APR) ^27^. Our practice and the standard of care for these patients is upfront treatment with chemoradiotherapy and observation for response for a minimum of 6 months with clinical exam and imaging. In the case of residual disease, an abdominoperineal resection with end colostomy is performed. A groin dissection should be added in patients with inguinal nodal disease ^22^. Long-term survival after salvage resection is possible, but this generally portends a poor prognosis. In recent retrospective series of patients undergoing salvage APR, 5-year survival ranged from 23% to 30% ^28– 30^. Overall, the evidence supporting the use of surgery is largely retrospective and consideration should be given to referral for clinical trials in these patients.

In recent years, interest in therapeutic vaccination has increased for epithelial neoplasms related to HPV, including anal cancer. Multiple approaches have been considered, though most therapeutic vaccines target the E6 and E7 oncoproteins. These vaccines attempt to deliver the E6/E7 antigen to antigen-presenting cells, leading to CD8 ^+^ cytotoxic T-cell activation ^31^. Multiple clinical trials are ongoing in this field ^32, 33^.

E6 and E7 can also be targeted through other means. A pilot study in women with metastatic cervical cancer used autologous T-cells selected for reactivity to HPV-E6 and -E7. Three of nine patients had a response, with two complete responses achieved at up to 22 months ^34^.

### Radiotherapy

Several recent advances have been made to reduce the toxicity associated with radiation. In addition to the obvious benefit to patients, reducing radiation complications can also improve disease control. Patients with a gap in radiotherapy, which is most often due to treatment toxicity, are known to have poorer local control of their disease ^35^. Technological advances have allowed the manner in which radiotherapy is delivered to evolve. Intensity-modulated radiation therapy (IMRT) is a technique in which the radiation dose is delivered in a highly precise, three-dimensional manner, limiting the dose to surrounding normal tissue such as the bone marrow ^36^. In two small prospective studies, the use of IMRT was associated with reductions in toxicity ^37, 38^. While there have been concerns that the increased complexity of IMRT can lead to a heightened need for vigilant quality control, excellent long-term outcomes have been reported with IMRT ^39, 40^.

## Special cases

### HIV infection

With the introduction of antiretroviral therapy (ART), patients with HIV and anal cancer can achieve outcomes equivalent to those seen in the general population ^41^. Since control of HIV infection with ART is paramount in achieving these outcomes, a multidisciplinary approach involving HIV care providers and pharmacists is particularly beneficial ^42^. There is some evidence that HIV-positive patients with a CD4 count of less than 200 may have less tolerance to chemoradiation ^43, 44^. However, ART can improve treatment tolerance ^45^. Overall, the care of HIV-positive patients should deviate from typical practice standards only when absolutely necessary.

### Other special populations

Some patients may require a reduction in the intensity of therapy that is undertaken. This is particularly true in elderly patients and those with a reduced functional status. Some success has been shown in elderly patients with the use of reduced-dose radiotherapy and 5-FU alone; however, this is often used in a palliative setting. In a retrospective series, this has been shown to have an acceptable side-effect profile ^46^. Modification of treatment may also need to be undertaken in patients who have a history of prior pelvic radiation. Our practice is still to refer such patients to radiation oncology and to a multidisciplinary tumor board to ensure that all treatment options have been exhausted. The ultimate treatment plan depends heavily on the individual patient’s history, prior treatment, and planned therapy.

### Anal melanoma

Anal melanoma is a rare malignancy with a dismal prognosis and a natural history quite different from that of anal squamous cell carcinoma ^47, 48^. Lesions may be pigmented and often present with bleeding. Metastatic disease is often present at diagnosis and is the major source of morbidity and mortality ^49^. Surgical resection is the treatment of choice for anal melanoma; however, the extent of resection is debated ^50^. Local excision should be attempted if an R0 resection is possible, particularly for symptomatic patients. There is emerging evidence that targeted therapies such as imatinib may be useful in select patients, although further research is needed ^51^.

### Anal adenocarcinoma

Anal adenocarcinoma is a rare malignancy of the anal canal ^52^. Owing to its rarity, the optimal treatment strategy for anal adenocarcinoma has not been established, there is very little evidence to guide treatment, and most are treated like low rectal cancers. Most patients undergo neoadjuvant chemoradiation followed by APR ^53, 54^.

## Conclusion

The incidence of anal cancer is gradually increasing. Fortunately, primary prevention via HPV vaccination is now widely available, and we are hopeful that rates will decline in the future. For most patients with anal cancer, cytotoxic chemotherapy and radiation remain the mainstays of treatment. In the near future, treatment with biological therapies, therapeutic vaccination, and immunotherapy is likely to improve outcomes in patients with anal cancer.
